# Extensive cervico-vaginal ischemic injury reaching the bladder base following obstructed labor in a macrosomic pregnancy: a case report

**DOI:** 10.1097/RC9.0000000000000062

**Published:** 2026-01-09

**Authors:** Malaka Abubakir, Ahmad Bishr Nasra, Lana Sabbagh, Rawia Barghouth, Majd Mohammad Al Ali

**Affiliations:** a Department of Medicine, Faculty of Medicine, University of Aleppo, Aleppo, Syrian Arab Republic; b Department of Medicine, Faculty of Medicine, Damascus University, Damascus, Syrian Arab Republic; c Department of Obstetrics and Gynecology, Faculty of Medicine, Tishreen University Hospital, Lattakia, Syrian Arab Republic

**Keywords:** bladder, case report, cephalopelvic disproportion, macrosomia, necrosis, obstructed labor

## Abstract

**Introduction:**

Fetal macrosomia, defined as a birth weight (BW) ≥4000 g, is associated with increased maternal and neonatal morbidity, particularly when BW exceeds 4500 g. While complications such as shoulder dystocia and postpartum hemorrhage are well recognized, extensive ischemic soft-tissue injury of the lower genital tract and adjacent urinary structures due to obstructed labor alone is exceedingly rare.

**Case Presentation:**

A 22-year-old gravida 3, para 2 woman at 40 weeks of gestation presented in active labor with signs of cephalopelvic disproportion, a prominent caput succedaneum, and profuse genital bleeding. Examination revealed severe ischemic–hemorrhagic injury and devitalization of the cervix and the anterior and posterior vaginal walls, extending to the level of the bladder base. Ultrasound confirmed fetal macrosomia and persistent occiput posterior position. An emergency cesarean section was performed, followed by a peripartum hysterectomy and partial vaginal wall resection due to extensive lower genital tract injury. The neonate was delivered in good condition with Apgar scores of 10 at both 1 and 5 min. Both mother and infant were discharged in stable condition the next day.

**Discussion:**

This case represents a rare instance of severe ischemic soft-tissue injury of the lower genital tract, consistent with necrosis, caused by prolonged obstructed labor in the setting of macrosomia, without prior surgical intervention. Sustained mechanical pressure during labor can compromise perfusion to pelvic tissues, resulting in life-threatening complications if not promptly addressed.

**Conclusion:**

Early recognition of obstructed labor and rapid surgical intervention are critical to prevent severe maternal morbidity in macrosomic pregnancies. This case highlights the importance of timely decision making in labor management to avoid irreversible complications.

## Introduction

Fetal macrosomia, defined as a birth weight (BW) of ≥4000 g, affects approximately 12% of newborns born to women without diabetes and 15–45% of those born to women with gestational diabetes mellitus (GDM)^[1]^. Macrosomia increases maternal risks, including emergency cesarean sections (CSs), postpartum hemorrhage (PPH), and obstetric anal sphincter injury (OASIS), while also posing even greater neonatal risks, such as shoulder dystocia and brachial plexus injury^[2,3]^. Severe macrosomia (BW > 4500 g) further elevates these risks^[4]^.

**HIGHLIGHTS**

- Prolonged obstructed labor can cause ischemic necrosis of pelvic soft tissues due to sustained mechanical compression.
- Fetal macrosomia significantly increases the risk of labor complications and maternal morbidity.
- Early recognition of soft tissue ischemia in labor is essential to prevent life-threatening outcomes.
- Severe ischemic necrosis may require radical surgical intervention such as hysterectomy.
- Timely surgical management improves maternal prognosis in cases of obstructed labor with tissue necrosis.

Uterine and vaginal necrosis are exceedingly rare and typically occur after interventions like uterine artery embolization or the application of B-Lynch sutures^[5,6]^. However, prolonged obstructed labor can itself lead to pressure necrosis of soft tissues, resulting in vesicovaginal or rectovaginal fistulae^[7]^. When membranes rupture early and cervical dilation stalls, sustained compression against the pelvic bones predisposes the patient to cervical and vaginal ischemic injury^[8]^.

Very few reports have described extensive ischemic injury of the uterine cervix and both anterior and posterior vaginal walls, extending to the level of the bladder base, caused solely by mechanical compression from a macrosomic fetus during obstructed labor. This report describes such a case, highlighting the need for early recognition of severe ischemic soft-tissue complications in the context of extreme macrosomia. This manuscript has been reported in line with the SCARE 2025 criteria^[9]^.

## Case presentation

A 22-year-old woman presented to our institution with complaints of profuse genital bleeding. She was gravida 3, para 2, aborta 0, at 40 weeks of gestation.

She lived in a rural area with limited access to formal healthcare and had not been enrolled in a structured antenatal care program. She reported only occasional visits to a local provider during pregnancy, and no written antenatal records were available. According to the patient, she had been informed that her “blood sugar was high” and advised dietary restriction, but no glucose tolerance test results or pharmacological treatment were documented. She had no known history of chronic hypertension or other chronic illness. Labor pains began spontaneously at home and, according to the patient and her family, continued with strong, regular contractions for many hours before she sought medical attention. She initially remained at home because home delivery is customary in her village, the nearest hospital is distant, and she was reluctant to be examined by male obstetricians; she presented only when the pain and bleeding became intolerable.

Upon admission, her vital signs were as follows: pulse 93 bpm and blood pressure 130/70 mmHg. A pelvic examination revealed active labor with a prominent caput succedaneum and evidence of cephalopelvic disproportion, with the presenting part remaining high despite strong contractions. Severe edema and bruising of the external genitalia were noted. On speculum and bimanual examination, the cervix could not be identified as a normal circumferential ring; instead, a friable, dark hemorrhagic mass was seen protruding into the upper vagina, with markedly congested anterior and posterior vaginal walls extending cranially towards the level of the bladder base. These findings were judged to represent severe ischemic–hemorrhagic injury and devitalization of the cervix and vaginal walls. Laboratory investigations were largely unremarkable, except for a hemoglobin level of 9 g/dl. Histopathological examination of the resected tissues was not available due to local resource limitations.

**Figure 1.**
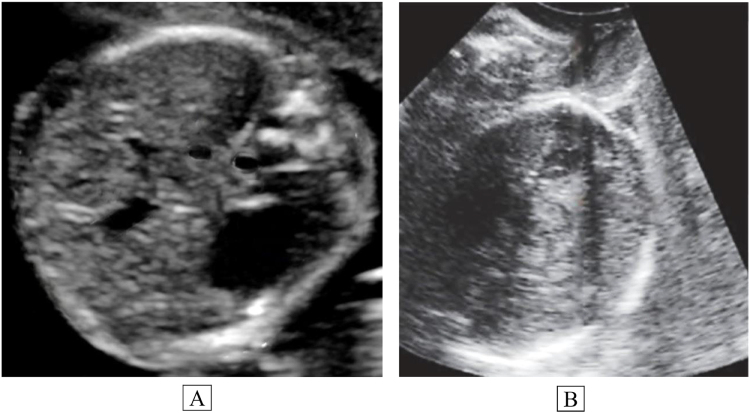
(A) Obstetric ultrasound at term showing a transverse section of the fetal head in cephalic presentation with an enlarged cranial diameter, consistent with suspected fetal macrosomia. (B) Obstetric ultrasound at term showing a longitudinal view of the fetal head deep in the maternal pelvis, with an enlarged cranial diameter consistent with suspected fetal macrosomia and cephalopelvic disproportion in labor.

Obstetric ultrasound indicated fetal macrosomia, which was considered the likely contributing factor to the observed soft-tissue injury. The ultrasound also confirmed failure to progress in labor due to cephalopelvic disproportion, as well as a right occiput posterior fetal position (Fig. 1).

**Figure 2.**
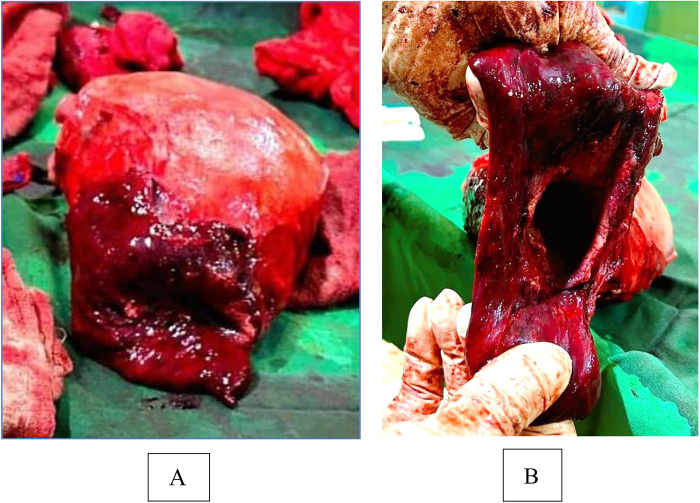
(A) Gross specimen of the uterus with attached cervix and proximal vagina following peripartum hysterectomy, showing extensive dark hemorrhagic and devitalized tissue involving the cervix and upper vaginal segment, consistent with severe ischemic–hemorrhagic injury after prolonged obstructed labor. (B) Resected lower genital tract specimen (cervix and vaginal walls) following peripartum hysterectomy, showing a thickened tubular segment with a central lumen and diffuse dark hemorrhagic, devitalized mucosa, consistent with severe ischemic–hemorrhagic injury after prolonged obstructed labor.

The patient underwent an emergency Caesarean section under spinal anesthesia. Due to the extent of lower genital tract injury and devitalized tissue, a peripartum hysterectomy was performed, along with partial resection of the vaginal walls (Fig. 2). Intraoperatively, the patient received three units of packed red blood cells, and adequate hemostasis was achieved. Intraoperative inspection confirmed intact ureters and bladder. Two surgical drains were placed, and a compression bandage was applied to the pelvic area.

Postoperatively, the patient remained hemodynamically stable, with a hemoglobin level of 10 g/dl and satisfactory urinary and gastrointestinal function. The infant had a BW of 4700 g and exhibited no immediate complications, with Apgar scores of 10 at both 1 and 5 min. Both the mother and infant were discharged in good condition after a short, uncomplicated postoperative hospital stay.

## Discussion

Fetal macrosomia, defined as a BW of ≥4000 g, affects a significant proportion of pregnancies and is strongly associated with GDM^[1,10]^ Several large cohort studies and meta-analyses have reported maternal complications associated with macrosomia, including emergency CS for fetal distress or failure to progress, PPH, and OASIS, as well as neonatal complications such as shoulder dystocia, brachial plexus injury, fractures, and birth asphyxia^[2-4]^.

The risk of complications is greater for the neonate than for the mother. In pregnancies with a BW >4000 g, the risk of emergency CS, PPH, and OASIS roughly doubles compared to non-macrosomic pregnancies, whereas in cases of severe macrosomia (BW > 4500 g), these risks increase approximately threefold^[2,4]^. The increase in neonatal risk is even more pronounced: for BW > 4000 g, the risk of shoulder dystocia, obstetric brachial plexus injury, and fractures rises several-fold, and for BW > 4500 g, these risks increase even more markedly^[2-4]^.

Prediction of fetal macrosomia relies on clinical assessment and ultrasonographic evaluation, with particular attention to maternal risk factors such as GDM, pregestational obesity, and excessive gestational weight gain^[1,10]^. Early identification of these risk factors allows for closer surveillance and tailored delivery planning with the aim of reducing adverse perinatal outcomes^[1,10]^.

This is a rare case of extensive ischemic injury of the uterine cervix and both anterior and posterior vaginal walls, extending to the level of the bladder base, due to mechanical compression by a macrosomic fetus during prolonged obstructed labor, necessitating hysterectomy in a patient without known predisposing iatrogenic risk factors.

Uterine necrosis is extremely rare; most reported cases occur after pelvic arterial embolization for PPH, embolization of a fibroid uterus, or application of B-Lynch sutures^[5,6,11]^. Prolonged obstructed labor can also result in vesicovaginal or rectovaginal fistula through pressure necrosis of soft tissue between the fetal head and the maternal pelvic bone, particularly in low-resource settings^[7]^. Prolonged obstructed labor, especially when membranes rupture early and cervical dilation stalls, remains a key risk factor for cervical and vaginal necrosis by sustaining compressive ischemia against the pelvic bones^[7,8]^.

Beyond the anatomical severity of this case, the clinical scenario highlights important gaps in antenatal and intrapartum care. In settings with structured antenatal follow-up, serial clinical and ultrasound assessments in women with suspected GDM or excessive fetal growth allow earlier recognition of macrosomia and enable timely counselling about the mode and place of delivery^[1,2,4,10]^. In our context, the absence of formal antenatal care, the patient’s rural residence, sociocultural preference for home birth, and reluctance to seek hospital care until pain and bleeding became intolerable all contributed to prolonged obstructed labor. Strengthening third-trimester surveillance, improving education about warning signs in labor, and facilitating accessible, female-staffed obstetric services in rural areas may help prevent similar catastrophic complications of macrosomic pregnancies.

Diagnosis is often made at the bedside: on speculum and bimanual examination, the cervix appears replaced by a hanging, discolored, friable mass, and the vaginal mucosa may show pale, sloughing areas indicative of necrosis.8 In contrast to congested or edematous tissue, which typically appears bluish-red and bleeds readily when touched, necrotic tissue is characteristically grey–black, malodorous, and poorly bleeding, with a sharp demarcation from adjacent viable areas^[7,8]^. These macroscopic features, together with the clinical history of prolonged obstructed labor, can strongly support a diagnosis of ischemic necrosis even in resource-limited settings where histopathological confirmation is not readily available^[7,8]^. In our patient, the cervix and vaginal walls appeared markedly hemorrhagic, thickened, and devitalized, with replacement of the normal cervical ring by friable tissue and extension towards the bladder base, consistent with severe ischemic soft-tissue injury of the lower genital tract. The absence of histopathological confirmation is a limitation and means that the degree of established necrosis cannot be precisely defined.

Hysterectomy is often the definitive treatment for uterine necrosis, particularly when conservative measures fail or the necrosis is extensive^[5,6,11]^. In select cases, conservative management without hysterectomy has been successful, especially when necrosis is limited and the patient’s condition is stable^[11]^. However, in our patient, the circumferential involvement of the cervix and the extensive injury of both anterior and posterior vaginal walls extending towards the bladder made uterine preservation unsafe, and peripartum hysterectomy with resection of the affected vaginal segments was the most appropriate life-saving option.

## Conclusion

This case illustrates a rare and severe complication of prolonged obstructed labor due to extreme fetal macrosomia, resulting in extensive ischemic soft-tissue injury and devitalization of the cervix and vaginal walls, extending to the level of the bladder base. Although uncommon, such outcomes underscore the critical importance of early recognition of labor dystocia and prompt intervention to prevent catastrophic maternal morbidity. Delayed diagnosis and management can lead to life-threatening hemorrhage, sepsis, irreversible lower genital tract damage, and the need for radical surgical procedures such as hysterectomy. Clinicians must maintain a high index of suspicion for ischemic complications in patients with signs of obstructed labor, particularly in the context of suspected macrosomia, to ensure timely and life-saving care.

## Data Availability

The datasets used during the current study are available from the corresponding author upon request.
